# Characterisation of male breast cancer: a descriptive biomarker study from a large patient series

**DOI:** 10.1038/srep45293

**Published:** 2017-03-28

**Authors:** Matthew P. Humphries, Sreekumar Sundara Rajan, Hedieh Honarpisheh, Gabor Cserni, Jo Dent, Laura Fulford, Lee B. Jordan, J. Louise Jones, Rani Kanthan, Maria Litwiniuk, Anna Di Benedetto, Marcella Mottolese, Elena Provenzano, Sami Shousha, Mark Stephens, Janina Kulka, Ian O. Ellis, Akinwale N. Titloye, Andrew M. Hanby, Abeer M. Shaaban, Valerie Speirs

**Affiliations:** 1 Leeds Institute of Cancer and Pathology, University of Leeds, Leeds, LS9 7TF, UK; 2 MD Anderson Cancer Centre, Houston, Texas, TX 77030, USA; 3 Department of Pathology, Bács-Kiskun County Teaching Hospital, Nyiri ut 38, H-6000, Kecskemet, Hungary; 4 Calderdale Hospital, Halifax, HX3 0PW, UK; 5 Surrey & Sussex NHS Trust, Redhill, Surrey, RH1 5RH, UK; 6 University of Dundee/NHS Tayside, Dundee, DD1 9SY, UK; 7 Barts Cancer Institute, Queen Mary University of London, London EC1M 6BQ, UK; 8 Department of Pathology and Laboratory Medicine, University of Saskatchewan, Royal University Hospital, Saskatoon, Saskatchewan S7N 0W8, Canada; 9 Poznan University of Medical Sciences, Greater Poland Cancer Centre, Poznan, 02-004, Warsaw Poland; 10 Department of Pathology, Regina Elena National Cancer Institute. Via Elio Chianesi 53, 00144 Rome, Italy; 11 Department of Histopathology, Addenbrooke’s Hospital, Cambridge, CB2 0QQ, UK; 12 Department of Histopathology, Imperial College Healthcare NHS Trust and Imperial College, Charing Cross Hospital, London W6 8RF, UK; 13 University Hospital of North Staffordshire, Stoke-on Trent, ST4 6QG, UK; 14 2nd Department of Pathology, Semmelweis University, Üllői út. 93, Budapest 1091, Hungary; 15 Faculty of Medicine & Health Sciences, Nottingham City Hospital. Nottingham, NG5 1PB, UK; 16 School of Medical Science, Kwame Nkrumah University of Science and Technology, Kumasi, Ghana; 17 Department of Cellular Pathology, Queen Elizabeth Hospital Birmingham and University of Birmingham, Birmingham, B15 2TW, UK

## Abstract

Male breast cancer (MBC) is rare. We assembled 446 MBCs on tissue microarrays and assessed clinicopathological information, together with data from 15 published studies, totalling 1984 cases. By immunohistochemistry we investigated 14 biomarkers (ERα, ERβ1, ERβ2, ERβ5, PR, AR, Bcl-2, HER2, p53, E-cadherin, Ki67, survivin, prolactin, FOXA1) for survival impact. The main histological subtype in our cohort and combined analyses was ductal (81%, 83%), grade 2; (40%, 44%), respectively. Cases were predominantly ERα (84%, 82%) and PR positive (74%, 71%), respectively, with HER2 expression being infrequent (2%, 10%), respectively. In our cohort, advanced age (>67) was the strongest predictor of overall (OS) and disease free survival (DFS) (p = 0.00001; p = 0.01, respectively). Node positivity negatively impacted DFS (p = 0.04). FOXA1 p = 0.005) and AR p = 0.009) were both positively prognostic for DFS, remaining upon multivariate analysis. Network analysis showed ERα, AR and FOXA1 significantly correlated. In summary, the principle phenotype of MBC was luminal A, ductal, grade 2. In ERα+ MBC, only AR had prognostic significance, suggesting AR blockade could be employed therapeutically.

With over 1.6 M cases diagnosed in females in 2012 alone, breast cancer is the most common cancer worldwide for women^1^. Significant research has been conducted into its biology and natural history over many decades which has helped in our understanding of its biology. Much less studied is male breast cancer (MBC) which accounts for around 1% of all breast cancers diagnosed^2^. For the UK and US this equates to approximately 350 and 2600 cases annually, respectively^3^,^4^. Both the US^5^ and the UK^6^ show a gradual increased incidence; 1.0 per 100 000 in the late 1970 s to around 1.2 per 100 000 at the start of this decade^7^.

The genesis of MBC is yet to be elucidated fully, with many studies suffering from small number of cases available, with published reports on as few 16 cases^8^. Information derived from these small numbers are at best anecdotal. In the last decade interest in MBC has grown, resulting in accumulation of more substantial numbers of cases allowing study of common biomarkers including estrogen receptor (ER) α, progesterone receptor (PR)^9^,^10^,^11^,^12^,^13^) and human epidermal growth factor receptor 2 (HER2)^9^,^10^,^11^,^12^,^13^ as well as less frequently evaluated biomarkers such as ERβ^9^ androgen receptor (AR)^9^,^10^,^12^,^13^,^14^), Bcl-2^10^,^13^, p53^10^,^12^,^13^, Ki67^10^,^11^,^13^,^15^, FOXP1^14^, GCDFP^14^, NAT1^16^, HLA^16^, MGB^14^, COX-2^17^, CD34^18^ and survivin^17^. BRCA mutations have also been studied^19^.

Through international collaboration, we accumulated a series of 446 MBC and evaluated and compared their clinicopathological characteristics with 15 published studies reporting ≥30 MBC cases. Using immunohistochemistry, we evaluated biomarkers with well-established roles in female breast cancer, represented on tissue microarrays (TMAs). These included ERβ1, ERβ2, ERβ5, PR, AR, Bcl-2, HER2, p53, E-cadherin, Ki67, survivin, prolactin and Forkhead box protein A1 (FOXA1). Our aim was to identify their expression and associate this with known clinical or pathological prognostic variables to determine potential prognostic roles in MBC.

## Methods

### Patients and ethical approval

Leeds (West) Research Ethics Committee granted ethical approval (06/Q1205/156). TMAs comprising 446 cases were constructed as previously described^9^. Between 2008 and 2010, cases from the UK (59%), Italy (11%), Canada (12%), Nigeria (2%), Hungary (9%) and Poland (7%) were collated into nine TMAs. Informed consent was not required as the anonymised material either pre-dated September 2006, or came from a Research Tissue Bank approved by the UK Human Tissue Authority (15/YH/0025). Under the terms of this project-specific ethics (06/Q1206/180), patient identities were not disclosed to the research team, hence specific informed consent was not required. Cases were pseudo-anonymised and data were analysed anonymously. Treatment details were not available for all cases. Where available, the majority received endocrine treatment.

### Immunohistochemistry

The antibody panel, selected given their relevance in female breast cancer, is shown in Table 1, alongside dilution and retrieval methods and cut-offs. Each biomarker was run as a batch with appropriate positive (tissue known to express the biomarker of interest) and negative (no primary antibody) controls. Immunohistochemistry was conducted as previously described^9^, employing REMARK criteria^20^. Briefly, heat-mediated epitope retrieval was achieved by pressure-cooking in 10% antigen retrieval Access Revelation 10X solution at 125 °C (Menarini Diagnostics, UK) or using 1% low pH antigen unmasking solution (Vector Laboratories, UK) for 2 minutes. Novolink^TM^ Polymer Detection System kit was used for visualization of primary antibodies following the manufacturer’s instructions (Leica Biosystems, UK). Slides were washed in TBST and stained in haematoxylin before dehydration in graded ethanol. Slides were mounted in DPX (Fluka, UK). Scoring criteria were selected according to previously reported studies (ER, PR, AR, ERβ1, ERβ2, ER β5^21^, FOXA1^22^, prolactin^23^, Ki67^24^, E-cadherin^25^, p53^26^, Bcl-2^25^, HER2^27^ and survivin^28^. TMAs were scanned (×20; Aperio ScanScope™) then manually scored using ImageScope™ viewing software (Aperio) by MPH and SSR, overseen by AMH and AMS, specialised consultant breast histopathologists.

### Biomarker associations

A correlation comparison was undertaken to visualise grouping of ERα, PR, AR and FOXA1 using Spearman correlation coefficient calculating pairwise combinations. A dataset containing scores for these biomarkers was uploaded to TMA Navigator^29^. Dendograms and correlation networks were generated providing abstraction of the relationships between multiple markers. Statistical significance was applied to identify minimum threshold values. (FDR P-value 0.05). Network significance was determined using algorithm AS89^30^. Benjamini–Yekutieli multiple hypothesis testing was applied.

### Statistical analysis

Associations with Disease-free and Overall survival (DFS; from initial diagnosis to the diagnosis of local or distant recurrence, OS; from initial diagnosis to death) were analysed (Kaplan–Meier plots, log rank test). Hazard ratios were determined by Cox regression. Follow up information was at least 10 years and was updated in June 2013 and survival periods calculated. Patients were censored at the last day they were known to be alive. Variables were entered in univariate and multivariate analysis (Cox proportional hazards regression model); these included the biomarker of interest, grade, nodal status and tumour size as is routinely used in analysis of breast cancer datasets. P values reported for univariate and multivariate analysis were calculated using Cox proportional hazards regression model in PASW (v21).

## Results

### Tumour characteristics

These are illustrated in Table 2, alongside studies published between 1996 and 2017, which have examined >30 MBCs. This information was collated to establish the spectrum of MBC data reported over the last 2 decades to affirm the representativeness of our cohort, with the combined average of all 1984 cases presented in the penultimate column of Table 2.

Comparatively our cohort characteristics are virtually identical to those from the combined data. The main histological subtype in our cohort and in the combined analyses was ductal (81%, 83%), grade 2 (40%, 44%), respectively. Lobular carcinoma was the second most common histology in both cohorts. The “other” category included cribriform, papillary, intraductal papillary, micropapillary, mucinous and DCIS. Cases were predominantly ER (84%, our cohort; 82% combined) and PR positive (74% our cohort; 71%, combined). HER2 expression was infrequent in our cohort (2%), and seen in 10% of cases from the combined cohort. Grade break down was very similar: grade 1 (11% our cohort; 13% combined), grade 2 (40%, our cohort; 44% combined), grade 3 (31%, our cohort 30%; combined). Despite missing/unavailable for grade (19%), node status (42%) and HER2 (33%) in our cohort, with the possible exception of node status, this was fairly typical of the other studies, illustrating the representativeness of our cohort. While this work was under review, data from the International Male Breast Cancer Pooling Project was reported^31^ and the characteristics of this cohort are presented for comparison in the final column of Table 2. Excluding missing data, this further emphasises the similarities of the datasets.

### Effect of tumour characteristics and biomarker expression on survival

#### Tumour characteristics

We performed univariate analysis on the whole cohort (n = 446) and in ERα + cases only (n = 375), considering the effects of grade, age, lymph node status and tumour size. This is shown in Table 3. Advanced age, was the strongest predictor of OS and DFS, both in the whole cohort (HR: 1.05 (1.03–1.08), p = 0.00001; HR: 1.04 (1.01–1.07), p = 0.01), respectively and in ERα+ cases only (HR: 1.07 (1.04–1.10), p = 0.000002; HR: 1.05 (1.01–1.08), p = 0.004), respectively. Node positivity was significantly associated with DFS in the whole cohort (HR: 1.91 (1.03–3.53), p = 0.04) and in ERα+ cases (HR: 2.72 (1.30–5.69), p = 0.008).

### Biomarkers

ERα, ERβ1, ERβ2, ERβ5, PR, AR, Bcl-2, HER2, p53, E-cadherin, Ki67, survivin, prolactin and FOXA1 expression were all evaluated (Table 3). In terms of cellular localisation, ERα, ERβ1, ERβ2, ERβ5, PR, AR, Bcl-2, p53, Ki67 and FOXA1 were predominantly nuclear, with characteristic membrane staining seen for HER2 and E-cadherin. Survivin displayed both nuclear and cytoplasmic staining. Examples are shown in Fig. S1.

**Figure 1:**
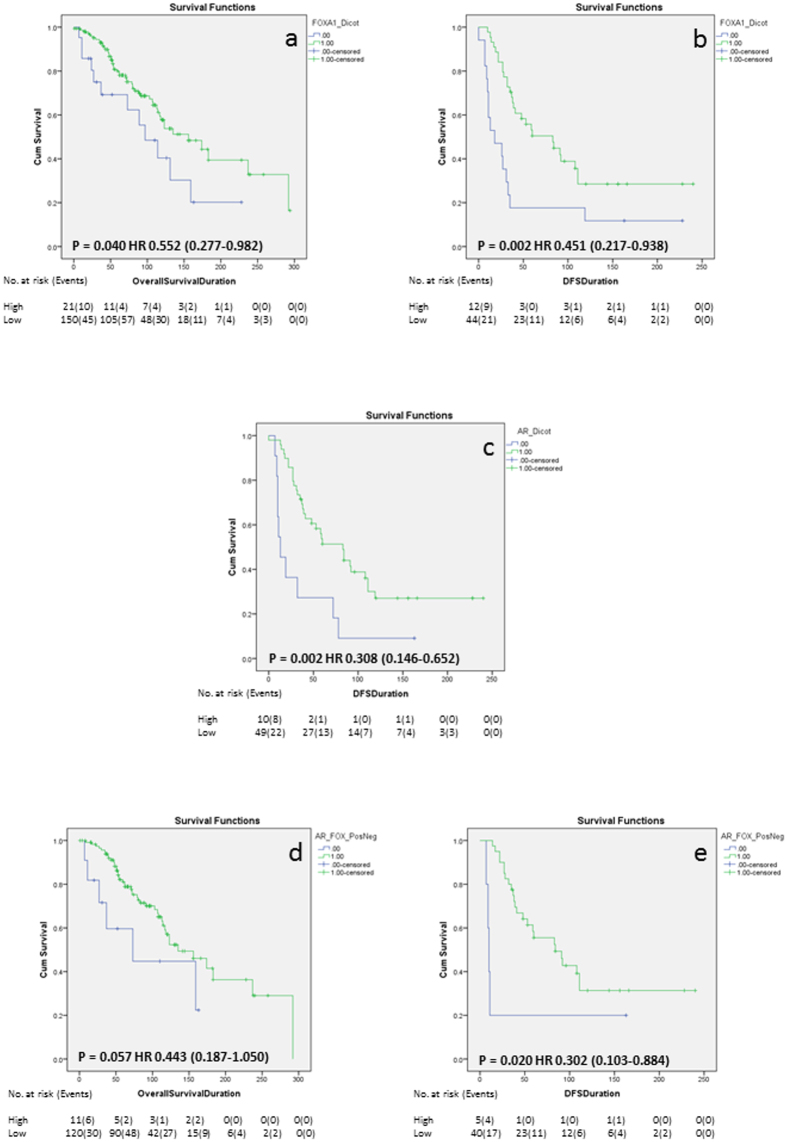
Kaplan-Meier survival analysis. Kaplan-Meier survival analysis showing the impact of FOXA1 expression on OS (**a**) and DFS (**b**), the effect of AR expression on DFS (**c**) and the impact of AR and FOXA1 co-expression on OS (**d**) and DFS (**e**). The number of at risk patients and events over time are displayed under each graph.

In the ERα+ cohort, Kaplan Meier survival analysis showed that FOXA1 was significantly associated with better OS and DFS (Fig. 1a and b, Log rank p = 0.04; 0.002, respectively), but did not remain upon multivariate analysis when adjusted for grade, age, size and nodal status (Table 4). AR was significantly associated with improved DFS only (Fig. 1c Log rank p = 0.002), that remained significant with multivariate analysis (Table 4; HR: 0.166 (0.04–0.56), p = 0.004). When evaluated in multivariate analysis with the addition of FOXA1, AR still remained independently significant (Table 4; HR: 0.205 (0.04–0.93), p = 0.04). None of the other biomarkers examined impacted on survival, and, in the case of survivin, was irrespective of its cellular location.

As FOXA1 is emerging as a critical player in breast cancer biology we examined the impact of its co-expression with AR and ERβ isoforms on survival in an ERα+ background. Here, co-expression of AR and FOXA1 (Fig. 1e; Log rank p = 0.02) was significantly associated with better DFS with a trend towards significance with OS for AR and FOXA1 (Fig. 1d; Log rank p = 0.06). ERβ5 and FOXA1 impacted positively on OS (Table 3; HR: 0.24 (0.05–1.01), p = 0.05) and DFS (Table 3; HR: 0.11 (0.02–0.68), p = 0.02). However, this was lost on multivariate analysis (not shown), where age still remained the strongest predictor of survival.

### Network inference using TMA Navigator

**Figure 2:**
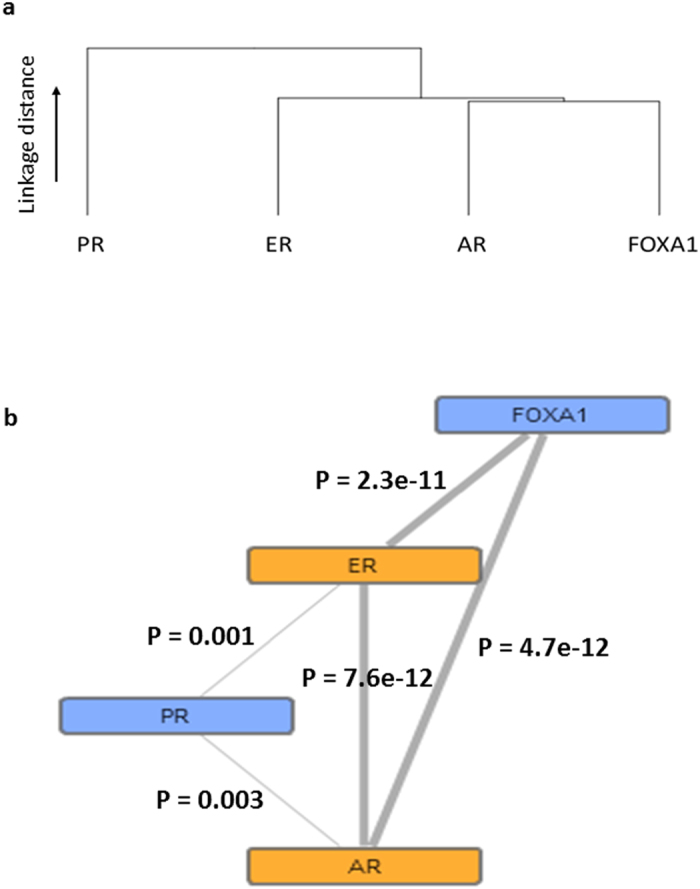
Correlative biomarker relationships. Dendrogram showing biomarker correlations using agglomerative hierarchical clustering with complete linkage (**a**) with an example protein network (**b**) showing significant correlation relationships indicated by the thickness of line connections. Nodes range from blue to orange, indicating low to high degree of significance, respectively. P values are displayed on the image.

Complete scores were available for PR, AR, and FOXA1 in 220 cases. A correlation comparison was undertaken to visualise the grouping of these biomarkers. The dendogram (Fig. 2a) demonstrates significant correlation between biomarkers using agglomerative hierarchical clustering with complete linkage. This identified ERα, AR and FOXA1 as being significantly correlated within our dataset (Fig. 2b). No association between PR and FOXA1 was observed.

## Discussion

Efforts towards better understanding the pathobiology of MBC are increasing with a number of groups, including our own, starting to accumulate sufficiently large numbers of cases to extend observations from purely anecdotal towards improving our knowledge of its underlying biology. Our international collaborative effort makes this one of the largest cohorts of MBCs examined to date, examining 446 cases and collating 1540 cases from published studies to identify common features of MBC. We do, however, acknowledge its limitations with regard to missing patient data and treatment information in some of the cases, despite our best efforts to obtain this.

Comparative analysis of clinicopathological data from our cohort with combined data extracted from 15 studies published over the last 21 years, reporting ≥30 cases^10^,^11^,^12^,^14^,^16^,^17^,^18^,^19^,^32^,^33^,^34^,^35^,^36^,^37^,^38^ was investigated totalling 1984 cases. Key observations from our cohort were that the majority of patients present with ductal histology, grade 2, with high incidence of ER and PR expression, reflective of luminal A phenotype. Nodal positivity was detected in approximately half of cases where this was known, with HER2 expression being much less frequent in all but one study, which reported almost 30% of cases as HER2 positive, which was confirmed by FISH^33^. Not only were these observations reflected in our cohort but they were also observed in the combined data from other studies excluding our own, which, with the exception of HER2, was very similar to our own cohort, and concurs with recent SEER data^39^. This highlights the representative nature of our cohort for further study. Furthermore, while this article was under review, the International Male Breast Cancer Program^40^ reported clinicopathological features of 1328 MBCs, with similar characteristics to our own (Table 2). We acknowledge a weakness of our study is the lack of complete information on node status in 41% of cases, despite out best efforts to obtain this. Nevertheless, missing node status was the most frequently under-represented variable in the studies outlined in Table 2, averaging 25% attrition, due to missing, unavailable or unreported data, especially in cohorts in excess of 200 cases, including those from the International Male Breast Cancer Program^16^,^31^,^33^.

The median age of our cohort was 67 years (range 30–97), in concordance with the average age of diagnosis of MBC, which is typically 10 years older than that seen for female breast cancer^2^. In this respect it is perhaps unsurprising that our analysis showed that advanced age was the strongest predictor of outcome, as reported in female breast cancer where those over 80 years of age had poorer survival, independent of their stage at diagnosis or the diagnostic period^41^. Nodal positivity and AR positivity were negatively prognostic for recurrence, in line with other works^9^.

As MBC is characteristically predominantly ERα-positive we evaluated the effect on outcome in ERα-positive cases only (84% of our cohort), investigating several biomarkers for their impact on survival. We found FOXA1 expression was positively prognostic for both OS and DFS. Often described as a pioneer factor, FOXA1 is emerging as a critical player in hormone dependent cancer, including breast^42^ and, in a meta-analysis, has been found to be significantly associated with ERα status in female patients^43^. FOXA1 correlates with survival duration in female breast cancer, where cases with high expression had significantly better survival^44^. Our results corroborate these findings for DFS in MBC. Although, this is the first time FOXA1 has been shown to positively impact survival duration in MBC, this was lost on multivariate analysis.

FOXA1 has the ability to bind to compacted chromatin, making these regions more accessible to other transcription factors, notably ERα and AR^45^. Since FOXA1 is a major determinant of ERα activity^42^, we assessed the impact of co-expression of FOXA1 with AR and ERβ isoforms. FOXA1 alone and AR expression alone was significantly associated with better DFS than ERβ5 alone. We have previously demonstrated the impact of ERβ5 in FBC survival^46^. While ERβ isoforms have been reported previously in MBC^9^, these did not impact on survival^9^. Interestingly, ERβ5 and FOXA1 co-expression showed a significant impact on DFS duration in MBC and showed a trend towards significance for OS.

FOXA1 typically works in cooperation with another transcription factor, GATA3. While we did not evaluate GATA3 in our work, a previous study has shown GATA3 positivity in only 6 out of 19 MBC (32%), compared to 82% of female breast cancer^47^. Furthermore GATA3 expression did not impact on survival in MBC, unlike FBC, where significantly increased mortality was observed. This suggests that the role of GATA3 may not be as important a transcription factor in MBC compared with female breast cancer, but further validation is required.

In the current study and previous work (which included 251 of the 446 cases evaluated in this study), AR expression was associated with better outcome in MBC^9^, for DFS but not OS, and this association remained upon multivariate analysis. AR is expressed across the main molecular subtypes of breast cancer and is gradually becoming recognised as a potential target for therapy in both genders^48^. Our results confirm these findings and could indicate potential use of anti-androgen therapy to treat MBC as demonstrated successfully in a recent report^49^.

We also identified a significant correlation of ERα, AR and FOXA1 expression using hierarchical clustering and correlation network analysis, with weaker, although still statistically significant, association with PR. We previously identified that ERα clustered with AR and ERβ in male but not female breast cancer which clustered with ERα and PR^9^.

In line with our findings, a previous study examining survivin in MBC showed no effect on survival, irrespective of its cell location^17^. However, none of the other biomarkers examined influenced outcome in MBC, despite showing significance in female breast cancer. This agrees partially with Kornegoor who showed no effect of Bcl2 on MBC survival but reported that p53 and HER2 were associated with poor survival^10^. Such disparity could be a reflection of cohort size, or potentially may suggest further differences in underlying biology between genders which is starting to be illuminated^9^.

In conclusion, the majority of MBC are luminal A, ductal grade 2 with nodal positivity in approximately half of all cases with HER2 expression being rare. While MBC expresses many of the same biomarkers as female breast cancer, of those examined, we found only AR remained significant upon multivariate analysis, providing potential for AR blockade to be employed therapeutically.

## Additional Information

**How to cite this article:** Humphries, M. P. *et al*. Characterisation of male breast cancer: a descriptive biomarker study from a large patient series. *Sci. Rep.* **7**, 45293; doi: 10.1038/srep45293 (2017).

**Publisher's note:** Springer Nature remains neutral with regard to jurisdictional claims in published maps and institutional affiliations.

## Supplementary Material

Supplementary Figure S1

## Figures and Tables

**Table 1:** Antibody dilutions and retrieval methods with scoring cut-offs for dichotomisation.

Antibody	Clone	Dilution	Incubation	Retrieval	Cut-Off
ERα	1D5	1:100	Overnight	High pressure heat retrieval in pressure cooker using 1% low pH antigen unmasking solution	Allred ≥ 2^21^
FOXA1	ab55718	1:500	Overnight		≥4^22^
PR	PgR 636	1:200	Overnight		Allred ≥ 2^21^
E- cadherin	NCH-38	1:100	Overnight		>50%^25^
Ki67	MIB1	1:100	Overnight		>14%^24^
p53	DO-7	1:1000	Overnight		>10%^26^
Bcl-2	124	1:200	Overnight		>10%^25^
HER2	PN2A	1:25	Overnight		2+ Confirmed by FISH^27^
Prolactin	B6.2	1:3000	Overnight		≥3^23^
Survivin	D8	1:25	Overnight		>5% (Nuc). ≥3 (Cyto)^28^
AR	AR441	1:100	Overnight		Allred ≥ 4^21^
ERβ1	MCA19745	1:20	1 hour	Access revelation	Allred ≥ 3^21^
ERβ2	MCA 2279	1:20	1 hour		Allred ≥ 3^21^
ERβ5	MCA 46764 A	1:50	1 hour		Allred ≥ 3^21^

**Table 2:** Comparison of clinicopathological features in MBC studies published since 1996 and examining >30 cases.

Feature	1This study	Study reference															Combined data excluding our study	Combined data including our study	Male Breast Cancer Pooling Project^31^
		12	11	10	14	32	16	33	19	34	17	18	35	36	37	38			
**Number**	446	111	91	134	73	145	220^2^	378	60	58	39	30	65	41	47	46	1538	1984	1328
**Histology**																			
** Ductal**	359 (81)	99 (89)	86 (95)	121 (90)	54 (74)	130 (90)	181 (82)	283 (75)	46 (77)	55 (95)	36 (92)	25 (83)	61 (94)	37 (90)	47 (100)	19 (41)	1280 (83)	1639 (83)	1123 (85)
** Lobular**	3 (1)	1 (1)	0 (0)	3 (2)	0 (0)	3 (2)	0 (0)	4 (1)	2 (3)	2 (3)	0 (0)	0 (0)	0 (0)	0 (0)	0 (0)	1 (2)	16 (1)	19 (1)	18 (1)
** Other**	63 (14)	10 (9)	5 (5)	10 (7)	19 (26)	12 (8)	0 (0)	13 (3)	12 (20)	0 (0)	3 (8)	5 (17)	4 (6)	4 (10)	0 (0)	7 (15)	104 (7)	167 (8)	155 (12)
** N/A**	21 (5)	1 (1)^+^	0 (0)	0 (0)	0 (0)	0 (0)	39 (18)^#^	78 (21)^#^	0 (0)	1 (2)^+^	0 (0)	0 (0)	0 (0)	0 (0)	0 (0)	19 (41)^#^	138 (9)	159 (8)	32 (2)
**Grade**																			
** 1**	48 (11)	14 (13)	2 (2)	32 (24)	14 (19)	35 (24)	15 (7)	33 (9)	2 (3)	7 (12)	25 (64)	1 (3)	9 (14)	1 (2)	8 (17)	4 (9)	202 (13)	250 (13)	292 (22)
** 2**	177 (40)	62 (56)	54 (59)	54 (40)	45 (62)	64 (44)	98 (45)	153 (40)	31 (52)	24 (41)		16 (53)	31 (48)	11 (27)	27 (57)	12 (26)	682 (44)	859 (43)	661 (50)
** 3**	137 (31)	26 (23)	26 (29)	48 (36)	10 (14)	46 (32)	85 (37)	79 (21)	27 (45)	13 (22)	13 (33)	13 (43)	25 (38)	29 (71)	12 (26)	8 (17)	460 (30)	597 (30)	359 (27)
** N/A**	84 (19)	9 (8) ^#^	9 (10)^+^	0 (0)	4 (5) ^$^	0 (0)	22 (10)^#^	113 (30)^#^	0 (0)	14 (24)^+^	1 (3)^+^	0 (0)	0 (0)	0 (0)	0 (0)	22 (48)^#^	194 (13)	278 (14)	16 (1)
**Node**																			
** **+	134 (30)	68 (61)	41 (45)	61 (46)	26 (36)	66 (46)	78 (35)	105 (28)	20 (33)	27 (47)	16 (41)	9 (30)	31 (48)	10 (24)	20 (43)	22 (48)	600 (39)	734 (37)	399 (30)
** −**	126 (28)	43 (39)	45 (49)	52 (39)	39 (53)	54 (37)	83 (38)	145 (38)	26 (43)	29 (50)	17 (44)	9 (30)	34 (52)	8 (20)	13 (28)	15 (33)	612 (40)	738 (37)	677 (51)
** N/A**	186 (42)	0 (0)	5 (5)^+^	21 (16)^+^	8 (11)^$^	25 (17)^+^	59 (27)^#^	128 (34)^#^	14 (23)^#^	2 (3) ^+^	6 (15)^+^	12 (40)^#^	0 (0)	23 (56)^#^	14 (30) ^#^	9 (20 ^#^	326 (21)	512 (26)	252 (19)
**ERα**																			
** **+	375 (84)	107 (96)	88 (97)	125 (93)	68 (93)	131 (90)	193 (88)	245 (65)	57 (95)	47 (81)	34 (87)	26 (87)	62 (95)	37 (90)	24 (51)	19 (41)	1263 (82)	1638 (83)	†
** −**	28 (6)	4 (4)	3 (3)	8 (6)	5 (8)	14 (10)	9 (4)	23 (6)	1 (2)	0 (0)	5 (13)	4 (13)	3 (5)	3 (7)	23 (49)	6 (13)	111 (7)	139 (7)	†
** N/A**	43 (10)	0 (0)	0 (0)	1 (1)^$^	0 (0)	0 (0)	18 (8)^#^	110 (29)^#^	2 (3)^#^	11 (19)^+^	0 (0)	0 (0)	0 (0)	1 (3)^#^	0 (0)	21 (46)^#^	164 (11)	207 (10)	†
**PR**																			
** +**	330 (74)	99 (89)	84 (92)	90 (67)	68 (93)	97 (67)	160 (73)	224 (59)	52 (87)	37 (64)	29 (74)	25 (83)	51 (78)	29 (71)	22 (47)	19 (41)	1086 (71)	1416 (71)	†
** −**	65 (15)	12 (11)	7 (8)	43 (32)	5 (7)	48 (33)	41 (19)	43 (9)	5 (8)	10 (17)	10 (26)	5 (17)	14 (22)	11 (27)	25 (53)	3 (7)	282 (18)	347 (18)	†
** N/A**	51 (11)	0 (0)	0 (0)	1 (1)^$^	0 (0)	0 (0)	19 (8)^#^	111 (29)^#^	3 (5)^#^	11 (19)^+^	0 (0)	0 (0)	0 (0)	1 (2)^#^	0 (0)	24 (52)^#^	170 (11)	221 (11)	†
**HER2**																			
** +**	7 (2)	9 (8)	13 (14)	4 (3)	1 (1)	5 (3)	18 (8)	55 (15)	5 (8)	2 (3)	4 (10)	3 (10)	6 (9)	18 (44)	0 (0)	7 (15)	150 (10)	157 (8)	†
** −**	290 (65)	102 (92)	72 (79)	130 (97)	68 (93)	140 (97)	157 (71)	139 (37)	50 (83)	17 (29)	35 (90)	27 (90)	56 (86)	22 (54)	0 (0)	10 (22)	1025 (67)	1315 (66)	†
** N/A**	149 (33)	0 (0)	6 (7)^+^	0 (0)	4 (5) ^$^	0 (0)	45 (21)^#^	184 (49)^#^	5 (8)^#^	39 (67) ^+^	0 (0)	0 (0)	3 (5)^#^	1 (2)^#^	47 (100)^$^	29 (63)^#^	363 (24)	512 (26)	†

^1^Includes data from Shaaban *et al*.^9^. ^2^Excludes frozen cases (n = 66). Due to lack of clear definition for grade and/or histological subtypes the analysis excludes refs 13, 15, 50, 51 and 52 which examined 77, 58, 30, 99 and 98 cases, respectively. Numbers in parentheses refer to percentages which were rounded to the nearest whole number. N/A refers to data which was not available^#^, missing^+^ or not reported^$^. ^†^These cases were stratified into molecular subtypes and hormone receptor status was not reported specifically.

**Table 3:** Univariate analysis of clinicopathological features and biomarkers with respect to ERα status.

Feature	Whole cohort (n = 446)				ERα+ cases (n = 375)			
	OS		DFS		OS		DFS	
	HR (CI)	P-value	HR (CI)	P-value	HR (CI)	P-value	HR (CI)	P-value
Grade	1.59 (1.01–2.51)	**0.05**	0.89 (0.48–1.66)	0.71	1.09 (0.73–1.61)	0.68	0.87 (0.55–1.37)	0.55
Age	1.05 (1.03–1.08)	**0.00001**	1.04 (1.01–1.07)	**0.01**	1.07 (1.04–1.10)	**0.000002**	1.05 (1.01–1.08)	**0.004**
Node positive	1.24 (0.75–2.08)	0.39	1.91 (1.03–3.53)	**0.04**	1.17 (0.68–2.01)	0.58	2.72 (1.30–5.69)	**0.008**
Size (>20 mm)	1.36 (0.75–2.47)	0.31	1.05 (0.34–2.54)	0.92	1.27 (0.69–2.33)	0.45	1.01 (0.41–2.48)	0.99
ER	1.00 (0.48–2.12)	0.99	1.12 (0.15–8.22)	0.91				
AR	0.86 (0.52–1.41)	0.55	0.39 (0.12–0.79)	**0.009**	0.84 (0.47–1.50)	0.57	0.30 (0.15–0.65)	**0.002**
ERβ1	1.15 (0.59–2.25)	0.68	0.91 (0.38–2.16)	0.82	0.87 (0.44–1.71)	0.69	0.91 (0.38–2.16)	0.83
ERβ2	1.14 (0.58–2.22)	0.71	1.40 (0.50–3.95)	0.52	1.25 (0.60–2.62)	0.56	1.37 (0.49–3.87)	0.55
ERβ5	0.91 (0.42–2.00)	0.82	0.38 (0.11–1.29)	0.12	2.39 (0.55–10.30)	0.24	0.38 (0.11–1.33)	0.13
FOXA1	0.75 (0.42–1.34)	0.33	0.41 (0.22–0.77)	**0.005**	0.55 (0.28–0.98)	**0.044**	0.45 (0.22–0.94)	**0.033**
PR	1.01 (0.57–1.81)	0.96	0.97 (0.43–2.19)	0.94	1.13 (0.57–2.26)	0.72	0.94 (0.39–2.24)	0.89
Survivin (nuclear)	1.07 (0.67–1.72)	0.782	0.96 (0.52–1.77)	0.89	1.05 (0.64–1.72)	0.86	1.03 (0.54–1.98)	0.93
Survivin (cytoplasmic)	0.76 (0.36–1.59)	0.47	0.61 (0.26–1.46)	0.27	0.48 (0.20–1.11)	0.07	0.55 (0.19–1.57)	0.26
Ki67	1.21 (0.72–2.04)	0.46	0.56 (0.27–1.17)	0.12	1.00 (0.57–1.86)	0.99	0.61 (0.28–1.30)	0.20
E-cadherin	1.56 (0.82–2.97)	0.18	0.54 (0.26–1.15)	0.11	1.47 (0.75–2.89)	0.26	0.54 (0.27–1.15)	0.11
Bcl-2	1.13 (0.41–3.12)	0.81	1.02 (0.31–3.38)	0.97	1.04 (0.33–3.35)	0.94	1.00 (0.30–3.33)	0.99
Prolactin	0.98 (0.61–1.59)	0.95	1.04 (0.57–1.88)	0.90	0.99 (0.60–1.65)	0.99	0.93 (0.49–1.79)	0.84
HER2	1.82 (0.25–13.22)	0.56	1.25 (0.30–5.27)	0.76	27.32 (3.19–233.88)	**0.003**	5.09 (0.63–41.40)	0.13
p53	0.97 (0.52–1.81)	0.91	0.45 (0.17–1.14)	0.09	0.95 (0.49–1.82)	0.87	0.45 (0.17–1.15)	0.09
AR+ FOXA1+	0.76 (0.35–1.62)	0.43	0.41 (0.16–1.09)	0.08	0.44 (0.19–1.05)	0.06	0.30 (0.10–0.88)	**0.03**
ERβ1+ FOXA1+	22.09 (0.04–11424))	0.33	1.31 (0.17–9.76)	0.79	20.86 (0.001–604504)	0.56	0.88 (0.21–3.83)	0.87
ERβ2+ FOXA1+	22.22 (0.06–7823)	0.30	21.84 (0.004–13372)	0.48	21.11 (0.005–82754)	0.47	21.84 (0.004–133724)	0.49
ERβ5+, FOXA1+	0.31 (0.09–1.06)	0.06	0.11 (0.02–0.68)	**0.02**	0.24 (0.05–1.01)	**0.05**	0.11 (0.02–0.68)	**0.02**

Significant P values are in bold. HR = hazard ratio, CI = confidence intervals.

**Table 4:** Multivariate analysis of clinicopathological features and biomarkers with respect to ERα status.

Feature	Whole cohort (n = 446)				ERα+ cases (n = 375)			
	OS		DFS		OS		DFS	
	HR (CI)	P-value	HR (CI)	P-value	1.22 (0.68–2.17)	0.50	0.93 (0.38–2.27)	0.87
Grade	1.58 (0.81–3.06)	0.18	1.51 (0.49–4.67)	0.47	1.07 (1.03–1.12)	**0.00008**	1.03 (0.98–1.07)	0.17
Age	1.06 (1.03–1.10)	**0.002**	1.04 (0.98–1.08)	0.13	1.09 (0.53–2.22)	0.81	2.81 (1.00–7.86)	**0.048**
Node pos	1.14 (0.57–2.27)	0.70	2.97 (1.04–8.48)	**0.042**	0.89 (0.43–1.83)	0.75	0.68 (0.24–1.91)	0.47
Size (>20 mm)	0.99 (0.49–1.99)	0.99	0.56 (0.18–1.71)	0.31	0.51 (0.14–1.78)	0.29	0.30 (0.05–1.73)	0.17
FOXA1	0.50 (0.17–1.48)	0.21	0.26 (0.05–1.37)	0.11	0.51 (0.14–1.78)	0.29	0.30 (0.05–1.73)	0.17
**Feature**	**Whole cohort (n** = **446)**				**ERα+ cases (n** = **375)**			
	**OS**		**DFS**		**OS**		**DFS**	
	**HR (CI)**	**P-value**	**HR (CI)**	**P-value**	1.20 (0.63–2.18)	0.54	0.61 (0.24–1.51)	0.28
Grade	1.78 (0.90–3.52)	0.09	0.98 (0.32–2.93)	0.97	1.08 (1.03–1.12)	**0.00008**	1.03 (0.99–1.07)	0.13
Age	1.07 (1.03–1.11)	**0.0001**	1.03 (0.99–1.07)	0.13	1.07 (0.52–2.18)	0.84	2.58 (0.89–7.49)	0.08
Node pos	1.10 (0.56–2.23)	0.78	2.78 (1.00–7.75)	**0.049**	0.95 (0.47–1.95)	0.90	0.71 (0.25–1.99)	0.52
Size (>20 mm)	1.06 (0.52–2.13)	0.87	0.58 (0.19–1.72)	0.33	1.11 (0.45–2.75)	0.81	0.14 (0.03–0.56)	**0.005**
AR	1.35 (0.54–3.36)	0.51	0.16 (0.04–0.56)	**0.004**	1.22 (0.66–2.18)	0.54	0.61 (0.24–1.51)	0.288
**Feature**	**Whole cohort (n** = **446)**				**ERα+ cases (n** = **375)**			
	**OS**		**DFS**		**OS**		**DFS**	
	**HR (CI)**	**P-value**	**HR (CI)**	**P-value**	1.29 (0.62–2.63)	0.48	1.43 (0.41–5.01)	0.568
Grade	1.76 (0.88–3.48)	0.10	1.02 (0.29–3.56)	0.97	1.07 (1.03 1.12)	**0.0002**	1.05 (0.99–1.12)	0.08
Age	1.06 (1.03–1.10)	**0.0002**	1.03 (0.99–1.08)	0.12	1.08 (0.48–2.39)	0.84	2.06 (0.66–6.39)	0.20
Node pos	1.08 (0.537–2.176)	0.82	2.85 (0.96–8.49)	0.05	1.23 (0.56–2.70)	0.59	0.56 (0.17–1.84)	0.34
Size (>20 mm)	0.99 (0.49–2.03)	0.99	0.57 (0.18–1.77)	0.33	0.42 (0.11–2.52)	0.42	0.20 (0.01–2.30)	0.19
AR	1.49 (0.57–3.86)	0.41	0.20 (0.04–0.93)	**0.04**	1.29 (0.63–2.63)	0.48	1.43 (0.41–5.01)	0.56
FOXA1	0.54 (0.15–1.96)	0.35	0.58 (0.08–4.08)	0.58	0.71 (0.20–2.51)	0.59	0.39 (0.08–1.91)	0.24

Significant P values are in bold. HR  = hazard ratio, CI = confidence intervals.
