# Supplementary material for: Characterisation of male breast cancer: a descriptive biomarker study from a large patient series
Source: Sci Rep. 2017 Mar 28;7:45293. doi: 10.1038/srep45293 (PMC5368596; doi:10.1038/srep45293)

**Characterisation of male breast cancer: a descriptive biomarker study from a large patient series**

Matthew P Humphries^1+^, Sree Sundara Rajan^1+^, Hedieh Honarpisheh^2^, Gabor Cserni^3^, Jo Dent^4^, Laura Fulford^5^, Lee Jordan^6^, J Louise Jones^7^, Rani Kanthan^8^, Maria Litwiniuk^9^, Anna Di Benedetto^10^, Marcella Mottolese^10^, Elena Provenzano^11^, Sami Shousha^12^, Mark Stephens^13^, Janina Kulka^14^, Ian O Ellis^15^, Akinwale N Titloye^16^, Andrew M Hanby^1^, Abeer M Shaaban^17^ and Valerie Speirs*^1^

^1^Leeds Institute of Cancer and Pathology, University of Leeds, Leeds, LS9 7TF, UK

^2^MD Anderson Cancer Centre, Houston, Texas, TX 77030. USA

^3^Department of Pathology, Bács-Kiskun County Teaching Hospital, Nyiri ut 38, H-6000,

^4^Calderdale Hospital, Halifax, HX3 0PW, UK

^5^Surrey & Sussex NHS Trust, Redhill, Surrey, RH1 5RH, UK

^6^University of Dundee/NHS Tayside, Dundee, DD1 9SY, UK

^7^Barts Cancer Institute, Queen Mary University of London, London EC1M 6BQ, UK

^8^Department of Pathology and Laboratory Medicine, University of Saskatchewan, Royal University Hospital, Saskatoon, Saskatchewan S7N 0W8, Canada

^9^Poznan University of Medical Sciences, Greater Poland Cancer Centre, Poznan, 02-004, Warsaw Poland

^10^Department of Pathology, Regina Elena National Cancer Institute. Via Elio Chianesi 53, 00144 Rome, Italy

^11^Department of Histopathology, Addenbrooke's Hospital, Cambridge, CB2 0QQ, UK

^12^Department of Histopathology, Imperial College Healthcare NHS Trust and Imperial College, Charing Cross Hospital, London W6 8RF, UK

^13^University Hospital of North Staffordshire, Stoke-on Trent, ST4 6QG, UK

^14^2nd Department of Pathology, Semmelweis University, Üllői út. 93, Budapest 1091, Hungary

^15^Faculty of Medicine & Health Sciences, Nottingham City Hospital. Nottingham, NG5 1PB, UK

^16^School of Medical Science, Kwame Nkrumah University of Science and Technology, Kumasi, Ghana

^17^Department of Cellular Pathology, Queen Elizabeth Hospital Birmingham and University of Birmingham, Birmingham, B15 2TW, UK


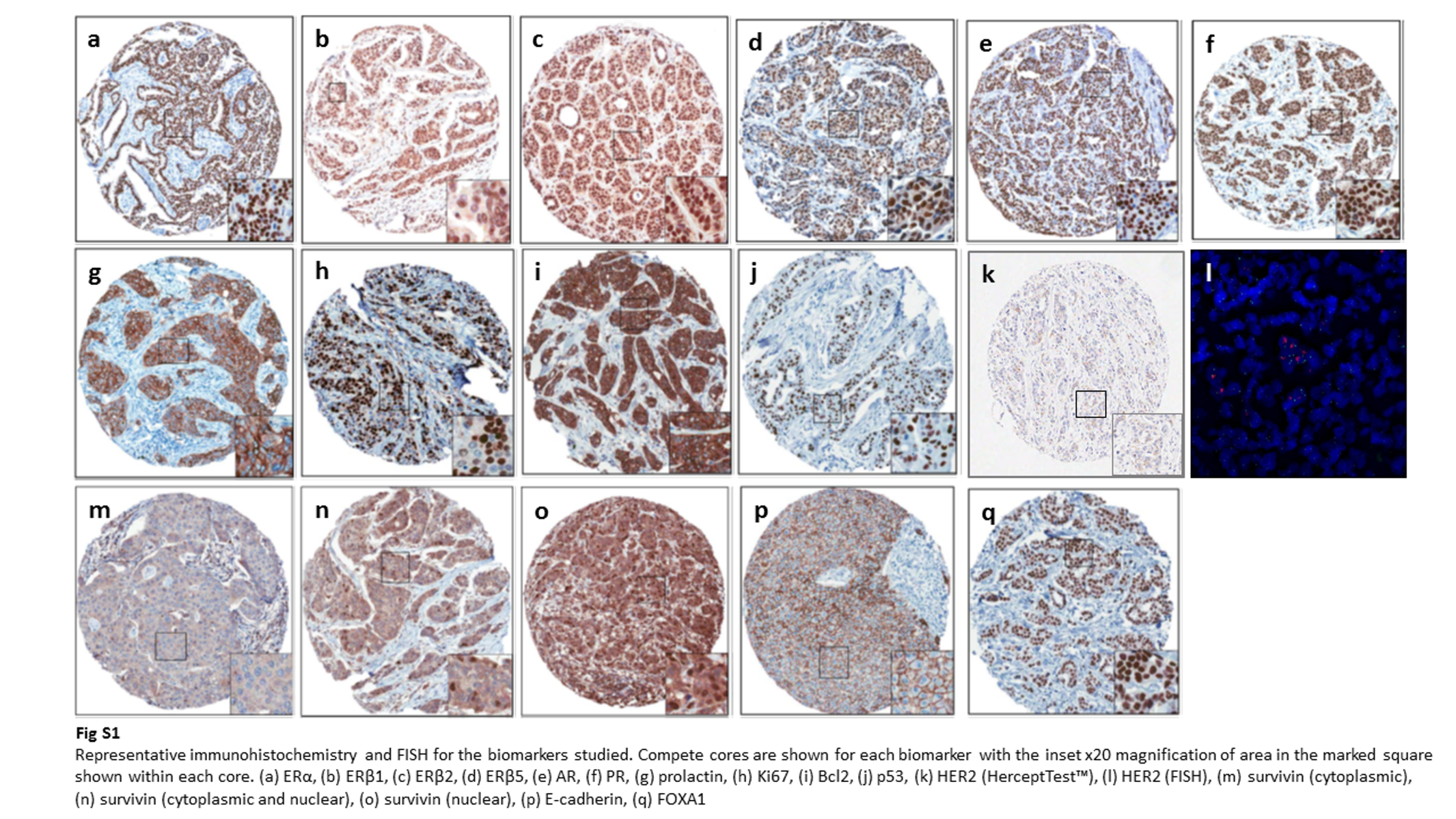

Supplement: Supplementary Figure S1 [file srep45293-s1.docx]
